# Genome-wide identification of markers for selecting higher oil content in oil palm

**DOI:** 10.1186/s12870-017-1045-z

**Published:** 2017-05-30

**Authors:** Bin Bai, Le Wang, May Lee, Yingjun Zhang, Rahmadsyah, Yuzer Alfiko, Bao Qing Ye, Zi Yi Wan, Chin Huat Lim, Antonius Suwanto, Nam-Hai Chua, Gen Hua Yue

**Affiliations:** 1 Temasek Life Sciences Laboratory, 1 Research Link, National University of Singapore, Singapore, 117604 Singapore; 2 R & D Department, Wilmar International Plantation, Palembang, Indonesia; 3 Biotech Lab, Wilmar International, Jakarta, Indonesia; 4 Bogor Agricultural University, Bogor, Indonesia; 5 Laboratory of Plant Molecular Biology, The Rockefeller University, New York, USA; 6 Department of Biological Sciences, National University of Singapore, Singapore, 117543 Singapore; 7 School of Biological Sciences, Nanyang Technological University, 6 Nanyang Drive, Singapore, 637551 Singapore

**Keywords:** RAD-seq, Mapping, QTL, Palm oil content, Oil yield

## Abstract

**Background:**

Oil palm (*Elaeis guineensis*, Jacq.) is the most important source of edible oil. The improvement of oil yield is currently slow in conventional breeding programs due to long generation intervals. Marker-assisted selection (MAS) has the potential to accelerate genetic improvement. To identify DNA markers associated with oil content traits for MAS, we performed quantitative trait loci (QTL) mapping using genotyping by sequencing (GBS) in a breeding population derived from a cross between Deli *Dura* and Ghana *Pisifera*, containing 153 F_1_ trees.

**Results:**

We constructed a high-density linkage map containing 1357 SNPs and 123 microsatellites. The 16 linkage groups (LGs) spanned 1527 cM, with an average marker space of 1.03 cM. One significant and three suggestive QTL for oil to bunch (O/B) and oil to dry mesocarp (O/DM) were mapped on LG1, LG8, and LG10 in a F_1_ breeding population, respectively. These QTL explained 7.6–13.3% of phenotypic variance. DNA markers associated with oil content in these QTL were identified. Trees with beneficial genotypes at two QTL for O/B showed an average O/B of 30.97%, significantly (*P* < 0.01) higher than that of trees without any beneficial QTL genotypes (average O/B of 28.24%). QTL combinations showed that the higher the number of QTL with beneficial genotypes, the higher the resulting average O/B in the breeding population.

**Conclusions:**

A linkage map with 1480 DNA markers was constructed and used to identify QTL for oil content traits. Pyramiding the identified QTL with beneficial genotypes associated with oil content traits using DNA markers has the potential to accelerate genetic improvement for oil yield in the breeding population of oil palm.

**Electronic supplementary material:**

The online version of this article (doi:10.1186/s12870-017-1045-z) contains supplementary material, which is available to authorized users.

## Background

Oil palm (*Elaeis guineensis*, Jacq.), mainly cultivated in the equatorial tropics of Africa, Southeast Asia and South America, is the world’s most efficient oilseed crop, as it produces about 25% of worldwide vegetable oil annually, 32% of global oils and fats output in 2012 and 40% of worldwide edible vegetable oil in 2014, while accounting for just 5.5% of global oil crop cultivation land use [1]. Palm oil is not only widely used in cooking and factories, but is also used for producing biofuels, which resulted in a continuous rise in demand for palm oil in recent years [2]. Planting higher yielding palms derived from improved germplasm genotypes is the most economically effective approach to guarantee adequate yield results in oil palm production. Using the *Tenera* genotype with thin shell (*sh*+, *sh*-), derived from crossing of *Dura* with thick shell (*sh*+, *sh*+) and *Pisifera* with no shell (*sh*-, *sh*-), resulted in a 30% increase in oil yield per hectare, hence improving the crude palm oil (CPO) yield in conventional breeding from 2.0 to 4.1 tons/ha in the last 50 years [1]. Recently, the global average CPO yield is 3.5 tons/ha of oil, still much lower than the estimated theoretical potential at 18 tons/ha [1]. Hence, there is substantial potential to increase CPO yield [3]. Although considerable genetic improvements have been made in yield in oil palm in the last several decades, the conventional improvement of oil palm is a complicated process, primarily due to long breeding cycles (i.e. over 20 years for males and 10 years for females per cycle) [1]. It is also difficult to select for complex traits such as oil content and oil quality. Marker-assisted selection (MAS) and genomic selection (GS) are expected to accelerate genetic improvement in breeding programs [4, 5].

QTL mapping refers to the identification of the molecular markers associated with quantitative traits on the whole genome [6]. It is the essential step towards MAS [4]. DNA markers, evenly covering a genome, are essential for QTL mapping. With the advent of next generation sequencing (NGS), single nucleotide polymorphisms (SNPs) are attractive DNA markers to use due to their high abundance, uniform distribution and compatibility with automated genotyping platforms [7]. A large number of DNA markers are essential for constructing of high-density genetic maps and fine mapping of QTL for important economical traits [7]. Restriction-site-associated DNA sequencing (RAD-seq) using NGS is an efficient approach to discover and genotype a large number of SNP markers [8, 9]. It has resulted in rapid and cost-effective massive marker discovery and has been successfully used to construct high-density linkage maps and fine QTL mapping in several plant species [10, 11]. In oil palm, several linkage maps have been constructed with simple sequence repeats (SSRs) markers and SNPs [12–16]. The genomes of *Elaeis guineensis* and another palm species *Elaeis oleifera* have been sequenced [17, 18], supplying the necessary reference genomes to facilitate genotyping by sequencing (GBS) [16]. In previous studies, QTL mapping has been conducted for shell thickness [12], bunch number (BN), fresh fruit bunch yield (FFB) and other yield traits [12, 13, 19], oil yield, oil to bunch content (O/B), fatty acid composition [20–24], plant growth, sex ratio [25], and embryogenesis in oil palm [26]. Also, a genome-wide association study (GWAS) identified three key loci for oil to dry mesocarp content (O/DM) in two palm populations of Deli × AVROS and Nigerian × AVROS in Malaysia [27]. However, the effects and locations of QTL identified for important traits in different palm populations are mostly different, suggesting that most QTL detected are population-specific. To facilitate MAS in oil palm, it is necessary to further identify and verify QTL for important traits in different genetic backgrounds.

Oil content traits (e.g. oil to bunch, oil to mesocarp, or oil to kernel) are important traits in oil palm production. Usually, oil yield is positively correlated with oil content in the fruits of oil palm [28]. The aim of this study was to construct a high-density linkage map and map QTL for O/B and O/DM in an oil palm breeding population generated from a cross of Deli *Dura* × Ghana *Pisifera*. The markers were identified using GBS. Using the markers closely linked with the significant QTL for oil content, the elite trees containing the QTL can be detected, which could accelerate improvement of oil yield in oil palm.

## Results

### Variation of oil content traits in a breeding population of *Dura* × *Pisifera*

The two oil content traits (i.e. O/B and O/DM) of the palm breeding population used for mapping QTL were evaluated for two and three harvest periods during 2011–2013, respectively. The average of the trait O/B was 30.18 ± 0.21% whereas the average of the trait O/DM was 79.23 ± 0.23% (Additional file 1: Table S1). A significant difference between individuals was found in the two traits (Additional file 1: Table S1, Additional file 2: Table S2). The coefficients of variation (CV) across different years or periods were 8.6–11.4% for O/B and 3.6–4.8% for O/DM (Additional file 1: Table S1). Statistical analysis of the phenotypic data showed that the two traits were normally distributed (Shapiro-Wilks test, *P* > 0.05) (Additional file 3: Figure S1), which indicates polygenic variation.

### Genotyping SNPs and SSRs in a breeding population

Five sequencing libraries containing 177 F_1_ trees from the population and two parents were constructed by double-digest RAD-seq approaches, and a total of 693.3 million clean reads were produced by the Illumina NextSeq 500 platform. After sequential quality filtering and sequence trimming, 20.7 and 17.7 million reads were produced for the two parents, and an average of 3.7 million clean reads were produced in each progeny. Using the data sets of the parental samples, a catalogue containing 80,113 loci was constructed. Then, the catalogue was used as reference for SNP discovery and genotyping in the mapping population using the program *genotypes* in Stacks software package [29]. After filtering out the markers with more than 20% missing data across all data sets, 2139 SNP markers were used for the mapping. From the mapping population, 24 out of 177 individuals were discarded for further analysis due to high missing data. All the SNP information of the remaining 153 palms, including map position and sequence, is shown in Additional file 4: Table S3. We also used a set of 150 microsatellites (SSRs) showing polymorphism between the two parental palms for genotyping the palm population. A total of 123 informative SSRs were used in the mapping population. The detailed information of SSRs, including map position and primer sequences, is shown in Additional file 4: Table S3. All the SNPs and SSRs were used to construct a high-density linkage map.

### Constructing a high-density linkage map and mapping the unassembled scaffolds of the genome in *Elaeis guineensis*

**Fig. 1:**
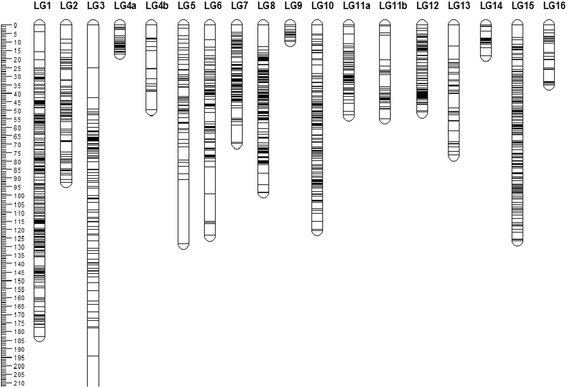
A high-density linkage map of oil palm (*Elaeis guineensis*) with 1357 SNPs and 123 SSRs The bar on left side represents the map length in cM, whereas the horizontal bars in each linkage group (LG) are positions of markers mapped

To construct a high-density linkage map and finer map QTL for important commercial traits, we developed and genotyped SNP markers using GBS by an improved RAD-seq approach [30]. The linkage map comprising 18 groups correspond to 16 LGs of previously published linkage map [13] were constructed for the *Tenera* population, of these LGs, two were each split into two smaller subgroups (LG4a, LG4b, LG11a, and LG11b). The linkage map, shown in Fig. 1, contains 1357 SNPs and 123 microsatellites, spans a total distance of 1527 cM and has an average marker space of 1.03 cM. The lengths of LGs ranged from 9.6 cM for LG9 to 221.5 for LG3, with an average of 95.4 cM. The marker intervals ranged from 0.46 cM on LG9 to 2.1 cM on LG5. In all LGs, except on LGs 3 and 5, all markers spaces were smaller than 20 cM. A summary of the markers, marker densities and genetic distances for each linkage group, and the relation of linkage groups corresponding to the draft genome [18] and the previous map [13], are shown in Table 1 and Additional file 4: Table S3.

**Table 1:** Summary of statistics of the linkage map of oil palm *Elaeis guineensis* Jacq

Linkage group	Markers	Length (cM)	Marker density (cM)	P2/Billotte^a^	Chromosome/Singh^b^	Size(bp)/Singh^b^
LG 1	257	182.6	0.71	LG 1	CHR 3	60,058,032
LG 2	76	92.3	1.21	LG 2	CHR 8	40,192,799
LG 3	116	221.5	1.91	LG 3	CHR 14	24,378,543
LG 4	49	66.5	1.36	LG 4	CHR 2	65,556,141
LG 5	61	128.3	2.10	LG 5	CHR 16	21,370,583
LG 6	85	123.4	1.45	LG 6	CHR 7	43,453,266
LG 7	95	69.4	0.73	LG 7	CHR 9	38,054,796
LG 8	154	98.3	0.64	LG 8	CHR 1	68,432,966
LG 9	21	9.6	0.46	LG 9	CHR 13	27,816,170
LG 10	139	120.5	0.87	LG 10	CHR 6	44,354,769
LG 11	89	107.7	1.21	LG 11	CHR 4	57,248,047
LG 12	99	51.2	0.52	LG 12	CHR 5	51,953,839
LG 13	39	76.5	1.96	LG 13	CHR 12	28,799,275
LG 14	20	18.0	0.90	LG 14	CHR 11	30,067,610
LG 15	154	126.4	0.82	LG 15	CHR 10	31,889,635
LG 16	25	34.8	1.39	LG 16	CHR 15	24,313,565

^a^Linkage map in oil palm published in 2005, Billotte et al. ^b^Oil palm genome sequencing in 2014, Singh et al.

**Fig. 2:**
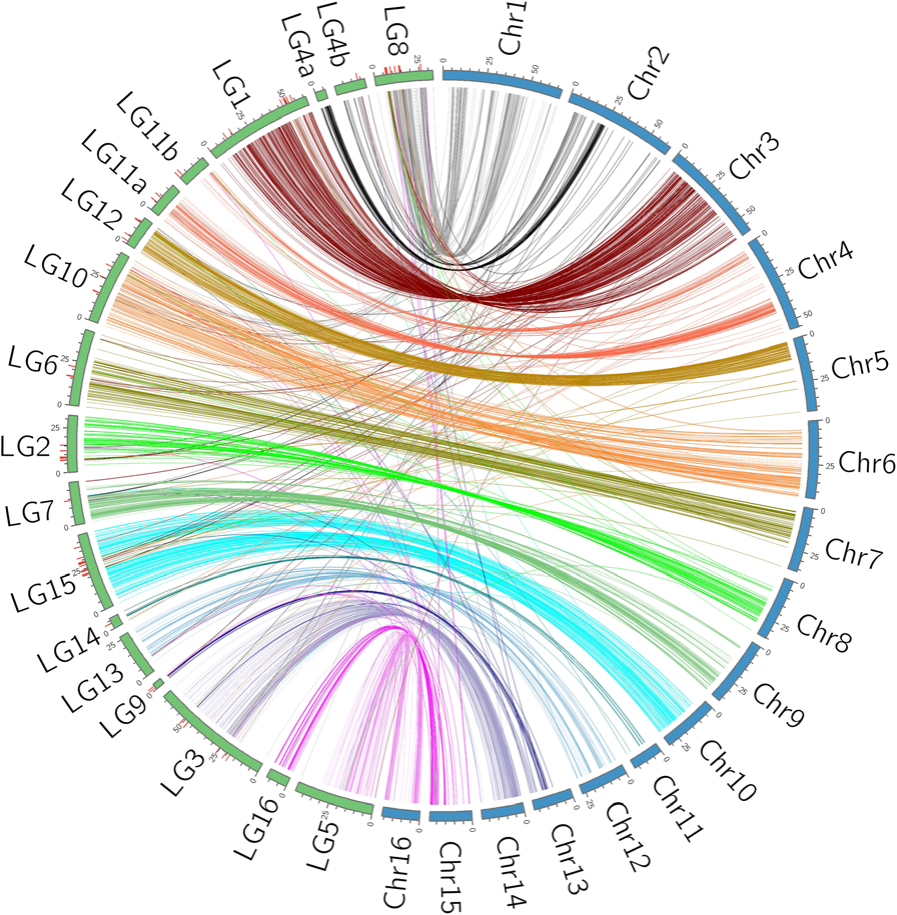
Synteny between genetic linkage groups and chromosomes of oil palm (*Elaeis guineensis*). The genetic linkage groups (LG) and the chromosomes (Chr) are represented by blocks coloured in green and *blue*, respectively. The short *red* lines outside the green LG blocks represent the contigs unassembled by reference genome. For each marker in this study, the corresponding chromosome and its physical position were determined by aligning the flanking marker sequences to the reference genome. The linkage groups were arbitrarily scaled so that the linkage group positions (cM) of the markers could be compared to their physical positions (bp) in the chromosomes

To facilitate the improvement of the assembly of the oil palm genome [18], we tried to map unassembled scaffolds to our genetic linkage map. In our high-density linkage map, 1357 SNPs and 123 microsatellites were assigned into 16 chromosomes. The sequences flanking 1345 markers were successfully aligned to the reference *Elaeis guineensis* genome. A total of 115 unassembled scaffolds (Additional file 4: Table S4) were successfully assigned to the 16 corresponding chromosomes. We investigated the genome syntenic relationships between LGs and chromosomes (Fig. 2). A high level of genomic synteny was observed between each LG and its corresponding chromosome across the whole genome of *Elaeis guineensis*. 93.8% of markers with known chromosome location were matched correctly from their linkage groups to the corresponding chromosomes (Fig. 2).

### Mapping QTL for oil content traits

**Fig. 3:**
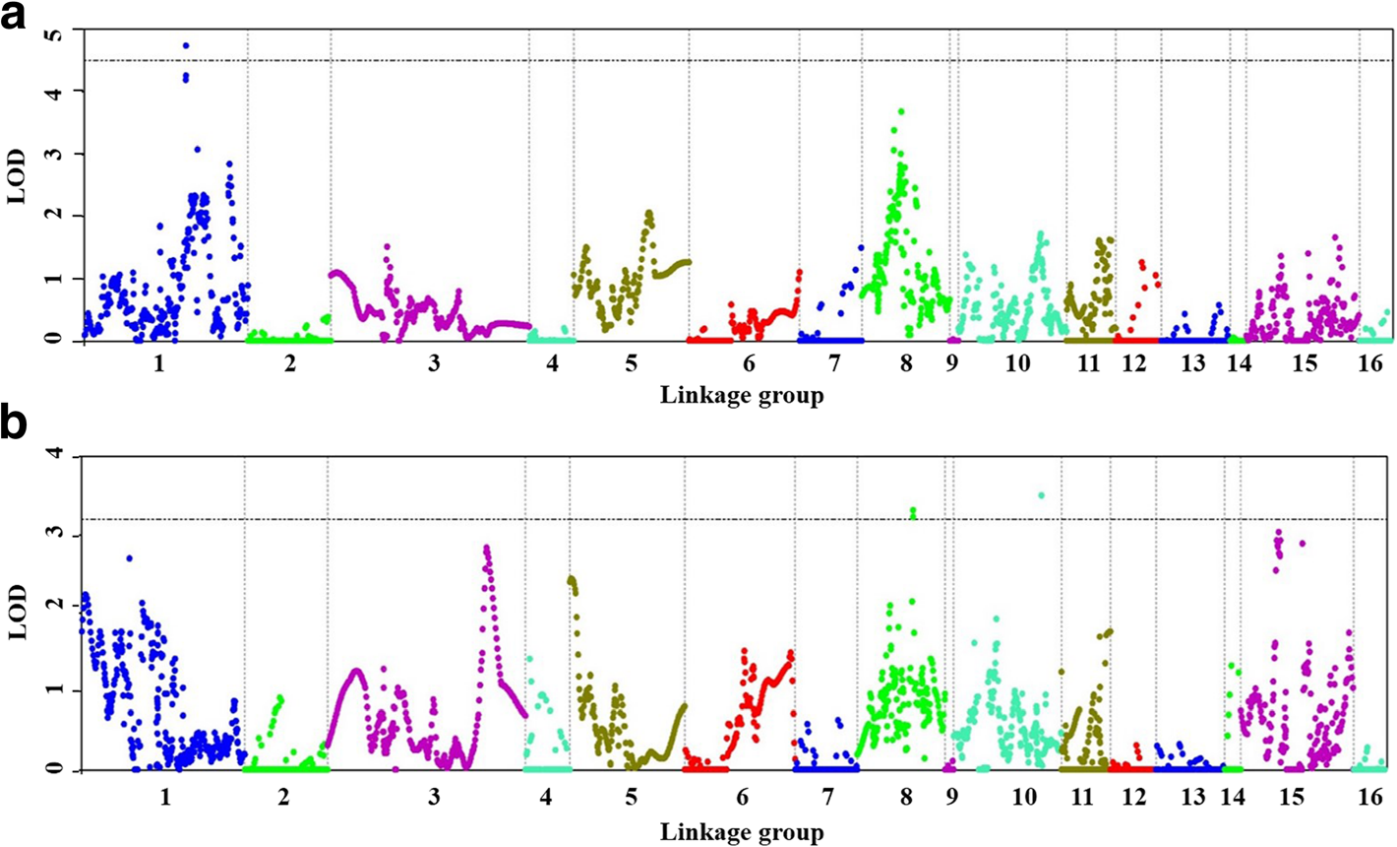
Whole genome scan of QTL for oil content traits in oil palm (*Elaeis guineensis* Jacq.). **a** QTL for averaged oil to bunch content (O/B) from 3 years, **b** QTL for averaged oil to dry mesocarp content (O/DM) from 2 years. The *dashed line* shows genome-wide significance at *P* = 0.05 (Figure **a**) and chromosome-wide significance at *P* = 0.05 (Figure **b**)

**Fig. 4:**
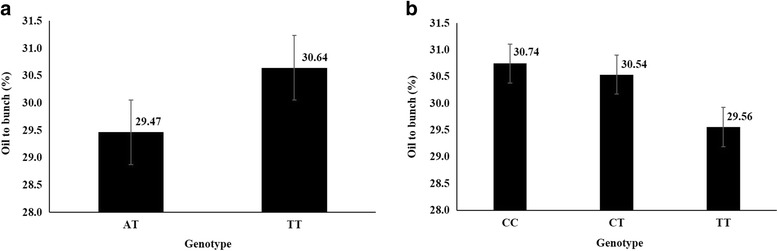
Differences of trait values between different genotypes at two SNPs flanking the QTL for average O/B in an oil palm breeding population **a** Average O/B at two different genotypes of marker EgSNP49153 from QTL region on LG1 **b** Average O/B at two different genotypes of marker EgSNP49181 from QTL region on LG1

By QTL mapping, one significant QTL *Qoil_bunch_1.1* for O/B, explaining up to 13.3% of phenotypic variance both in the first period and for averaged O/B among the three periods, was detected on LG1. Furthermore, one suggestive QTL *Qoil_bunch_8.1* for O/B on LG8 and two suggestive QTL (*Qoil_mesocarp_8.1* and *Qoil_mesocarp_10.1*) for O/DM on LG8 and LG10 were also identified both in the first period and for averaged value among the multiple periods in this study, explaining 10.5, 13.0 and 7.6% of phenotypic variance, respectively (Fig. 3 and Table 2). The confidence intervals of the four QTL were 1.92, 1.18, 0.92, and 0.14 cM (*P* < 0.05), respectively. The markers EgSNP49135 and EgSNP49181 flanked the significant QTL *Qoil_bunch_1.1*. The two genotype calls AT and TT of EgSNP49135 had average O/B of 29.47 and 30.64%, respectively, and the three genotypes CC, CT, and TT of EgSNP49181 had average O/B of 30.74%, 30.54%, 29.56%, respectively (Additional file 5: Table S5), hence showing significant differences in O/B in the breeding population (Fig. 4, *P* < 0.001). The LOD value and the nearest SNPs to the peak of each QTL region are listed in Table 2.

**Table 2:** Summary of QTL for oil content traits identified in the palm population of *Dura* × *Pisifera*

Trait	QTL	Linkage group	QTL interval^a^ (cM)	LOD Threshold^b^	LOD at peak	PVE^c^ (%)	The nearest linkage marker^d^
oil to bunch_1st period	*Qoil_bunch_1.1*	LG1	113.1–115.0	4.0	4.6	13.0	EgSNP49153*****
	*Qoil_bunch_8.1*	LG8	44.1–45.3	3.3	2.7	7.9	EgSNP61696**
oil to bunch_average	*Qoil_bunch_1.1*	LG1	113.1–115.0	3.8	4.7	13.3	EgSNP49153***
	*Qoil_bunch_8.1*	LG8	44.1–45.3	3.4	3.7	10.5	EgSNP61696******
oil to dry mesocarp_1st period	*Qoil_mesocarp_10.1*	LG10	98.1–99.0	3.5	3.5	10.0	EgSNP40659*
	*Qoil_mesocarp_8.1*	LG8	62.5–62.6	3.2	3.3	9.5	Eg0129*
oil to dry mesocarp_average	*Qoil_mesocarp_10.1*	LG10	98.1–99.0	3.5	4.6	13.0	EgSNP40659*
	*Qoil_mesocarp_8.1*	LG8	62.5–62.6	3.4	3.4	7.6	Eg0129*

^a^significant confidence intervals (*P* < 0.05), ^b^chromosome-wide LOD threshold, ^c^percentage of the phenotypic variance explained at the QTL, ^d^significance from Kruskal-wallis analysis by software MapQTL6

*: *P* < 0.05, **: *P* < 0.01, ***: *P* < 0.005, ****: *P* < 0.001, *****: *P* < 0.0005, ******: *P* < 0.0001

### The effects of QTL combinations on the increase of oil content

**Fig. 5:**
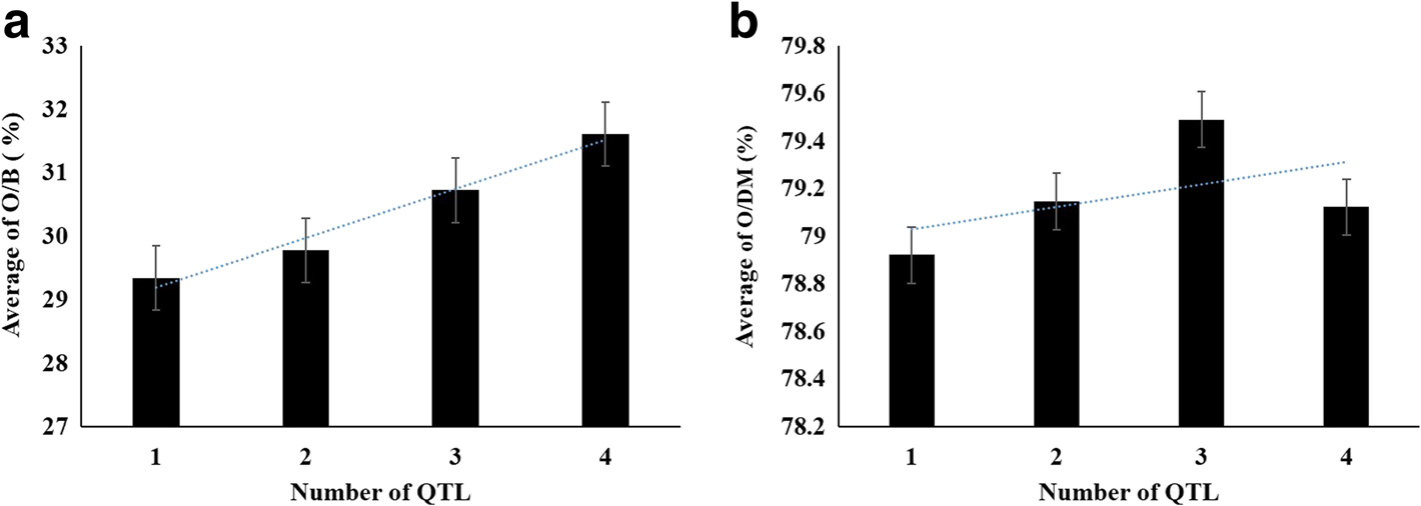
Effect of the combinations of beneficial marker genotypes at four QTL for oil content on the increase of oil content in a *Dura* × *Pisifera* breeding population of oil palm (*Elaeis guineensis* Jacq.) **a** Effect of the combinations of QTL with beneficial marker genotypes on the increase of oil to bunch content (O/B). **b** Effect of the combinations of QTL with beneficial marker genotypes on the increase of oil to dry mesocarp content (O/DM). The numbers 1, 2, 3, and 4 represent number of the individuals containing the QTL number with beneficial genotypes. 1: individuals with one of *QTL Qoil_bunch_1.1*, *Qoil_bunch_8.1*, or *Qoil_mesocarp_8.1*, respectively. 2: individuals with QTL combinations *Qoil_bunch_8.1* + *Qoil_bunch_1.1*, *Qoil_mesocarp_10.1* + *Qoil_bunch_1.1*, *Qoil_mesocarp_10.1* + *Qoil_bunch_8.1*, *Qoil_mesocarp_8.1* + *Qoil_bunch_1.1*, *Qoil_mesocarp_8.1* + *Qoil_bunch_8.1*, and *Qoil_mesocarp_8.1* + *Qoil_mesocarp_10.1*, respectively. 3: individuals with QTL combinations *Qoil_mesocarp_8.1* + *Qoil_mesocarp_10.1* + *Qoil_bunch_1.1*, *Qoil_mesocarp_8.1* + *Qoil_mesocarp_10.1* + *Qoil_bunch_8.1*, *Qoil_mesocarp_8.1* + *Qoil_bunch_8.1* + *Qoil_bunch_1.1*, and *Qoil_mesocarp_10.1* + *Qoil_bunch_8.1* + *Qoil_bunch_1.1*, respectively. 4: individuals with QTLs combinations *Qoil_bunch_8.1* + *Qoil_bunch_1.1* + *Qoil_mesocarp_8.1* + *Qoil_mesocarp_10.1*

To elucidate the effect of different QTL combinations on the increase of oil content in the palm breeding population, the association with phenotypic data of marker genotypes in four QTL was analysed: *Qoil_bunch_1.1*, *Qoil_bunch_8.1*, *Qoil_mesocarp_8.1*, and *Qoil_mesocarp_10.1*. The average O/B of individual palms containing one or two O/B QTL with beneficial genotypes were 30.35 and 30.97%, respectively, which was significantly higher than that of palms without those QTL with beneficial genotypes (average O/B was 28.24%) (*P* < 0.001). However, there was no significant difference between those having one and two O/B QTL with beneficial genotypes. The average O/B of individuals containing three or four QTL with beneficial genotypes for both O/B and O/DM were 30.72 and 31.60%, respectively. The average O/B of individuals containing any one QTL with beneficial genotype was 29.34%. The average O/B of individuals containing three or four QTL with beneficial genotype was significantly higher than those individuals containing just one QTL with beneficial genotypes (*P* < 0.05), and the more QTL with beneficial genotype contained in the palm individuals, the higher the average O/B (Fig. 5a). On the other hand, the differences in average O/B among palm individuals containing two to four QTL with beneficial genotype were not significant.

As for O/DM, the average phenotypic values were 79.35, 78.59, and 79.67% among individuals containing beneficial genotypes of *Qoil_mesocarp_8.1*, *Qoil_mesocarp_10.1*, and both *Qoil_mesocarp_8.1* and *Qoil_mesocarp_10.1*, respectively. These three groups had no significant difference between one another, but all had almost significant higher average O/DM values than those without any O/DM QTL with beneficial genotype (Fig. 5b). Similarly, there was no significant difference in O/DM among individuals containing two to four QTL with beneficial genotypes for both O/B and O/DM but these individuals all had higher average O/DM (79.14%, 79.49%, 79.12%, respectively) than those containing just one QTL with beneficial genotype (78.92%) (Fig. 5b).

## Discussion

### Genotyping by sequencing (GBS) for the heterozygous oil palm breeding population

With the rapid advancement in next generation sequencing technologies, together with cost decrease, GBS is now feasible for high-throughput SNP discovery and genotyping [8]. The ddRAD-seq with *Pst*I-*Msp*I used here for genotyping was an effective GBS method to get a large number of SNPs in plants [9, 31]. We used 48 bar codes and 12 index (adapter) sets for the Illumina NextSeq 500 platform, which produced paired-end raw reads of 2 × 150 bp in length. The GBS approach has been used firstly in a F_2_ population of Asian seabass for genotyping, which produced more than 18,000 SNPs across all the samples, and finally 3928 SNPs were genotyped in more than 80% of progenies [30]. Compared to the ~700 Mb Asian seabass genome, oil palm has a huge genome ~1.8 Gb [18]. We genotyped a F_1_ breeding population of *Tenera* palms derived from crossing of *Dura* and *Pisifera* using the same GBS approach. Only 2139 SNPs were genotyped in more than 80% of the palm progenies. A similar low number of SNPs genotyped was reported in another study on GBS in an oil palm F_2_ population derived from crossing *Dura* and *Pisifera* [16]. The lower number of SNPs genotyped in oil palm may be because the *Tenera* individuals, being derived from crossing two different forms, have a high heterozygosity in the genome, resulting in a lower digest efficiency of endonuclease and hence less common SNPs, than for crosses of the same forms or varieties. In the future, different endonucleases should be tested during the construction of ddRAD-seq libraries to improve the efficiency of GBS.

### The high-density linkage map and improved genomic assembly for unmapped scaffolds in *Elaeis guineensis*

In our study, we have constructed a high-density linkage map for oil palm, with 1480 DNA markers. The marker density was down to 1.0 cM in the intraspecific genetic map, from the previous 1.26 cM [16] and 1.4 cM [14] for the *Dura* and *Pisifera* intraspecific integrated maps, respectively. In previous studies on linkage mapping in oil palm, linkage maps were constructed with 252 to 944 genetic markers, including RAPD, SSRs, and AFLPs [13–15, 20]. However, AFLP and RAPD markers are mainly dominant markers, and SSRs confer lower throughput and are more laborious to use than SNP. In addition, these previous linkage maps of oil palm had a limited marker number due to few numbers of markers available and small oil palm population. The development of 200 k SNPs arrays and GBS technologies has enabled the genotyping of a large number of SNPs in larger oil palm populations, and thus the construction of high-density linkage maps [9, 27, 31]. In oil palm, a linkage map containing 1085 SNPs was constructed using GBS [16]. To the best of our knowledge, our linkage map is the densest linkage map of oil palm so far. The high-density linkage map will supply an essential tool for fine mapping QTL for MAS to accelerate genetic improvement of important traits. However, we noticed that on LGs 3 and 5, in a few positions, marker spaces were bigger than 20 cM, more markers should be mapped in these positions to reduce marker interval for QTL mapping. And two LGs (LG4, LG11) contained four small subgroups (LG4a, LG4b, LG11a, and LG11b) in this study, which could be due to the small number of markers called in the two LGs. One way to improve the marker density is to map the scaffolds of the sequenced genome [18] and re-sequenced genomes [17] to the linkage map using in-silico mapping [32]. Using our high-density genetic map, 115 unassembled scaffolds from the sequenced oil palm genome [18] were successfully assigned to the 16 corresponding chromosomes (Additional file 4: Table S4), which improved the genome assembly of oil palm. Alignment of 1345 markers mapped in our linkage map to the reference genome revealed a high level of genomic synteny between each LG and its corresponding chromosome across the whole genome of *Elaeis guineensis*. However, we noticed that 6.2% of aligned segments located on 16 chromosomes were not syntenic into corresponding linkage groups. Some of these could be caused by recombination in the mapping population, and some could be caused by incorrect genotyping or annotation in reference *Pisifera* genome sequence. Further improvement of the linkage maps by genotyping more DNA markers in larger mapping populations may improve the assembly of oil palm genome.

### QTL mapping for oil content related traits in *Elaeis guineensis*

O/B, O/DM and FFB yield are important components of oil yield in oil crops. Simultaneously increasing both traits is difficult to achieve in practical breeding programs because of the complex interdependence between plant developmental traits and yield components. On the other hand, oil content in fruit traits has generally high heritability in oil crops [33–35], and is also a complex quantitative trait that is directly related to oil yield in oil palm production [28]. We identified four QTL for O/B and O/DM averaged among all the periods in the breeding population. The four QTL were found on LG1, LG8, and LG10 in a *Tenera* population generated by crossing Deli *Dura* and Ghana *Pisifera*, respectively. Due to the variation of environments (different harvest periods), the phenotypic data of O/B and O/DM were significantly different in different harvest periods (*P* < 0.001, Additional file 2: Table S2), thus a significant environmental effect on the population can be inferred. The four QTL mapped in the population were for one harvest period and mean O/B and O/MD from three and two harvest periods, respectively.

Only a few QTL for oil related traits had been identified in these LGs in previous studies [21, 27, 36]. There were some differences in location as well as phenotypic effect between our QTL and other previously reported QTL. In these known QTL, the QTL on LG1 for oil yield/palm/year, palm oil to pulp ratio [36], and O/DM [27], were different from *Qoil_bunch_1.1* because of different traits and genomic location. The previous QTL for O/DM on LG10 was ~21 Mbp away from *Qoil_mesocarp_10.1* based on the physical position of the former’s linkage marker mEgCIR3826 on the genome of oil palm [21], and the QTL for O/DM on LG10 was ~27 M bp away from *Qoil_mesocarp_10.1* based on the oil palm genome [27]. Furthermore, we used the three key SNPs on chromosome 5 (corresponding to LG12) associated with O/DM detected by GWAS [27], to genotype two parental trees (Deli *Dura* and Ghana *Pisifera*) by Sanger sequencing in our mapping population. Unfortunately, two SNPs (TT genotype for SD_SNP_000010418, and CC genotype for SD_SNP_000002370) were not polymorphic in our mapping population, while the other (SD_SNP_000019529) were polymorphic, but not informative (i.e. TT genotype in Deli *Dura* and CC genotype in Ghana *Pisifera*). Therefore, in our population, we were not able to know the effects of these three SNPs. Compared to the previous studies on mapping of QTL for oil yield related traits in oil palm, the differences in the locations and effects of QTL for oil related traits suggest that the four QTL identified in this study could be novel loci and specific to our breeding population. All these data on QTL for oil content traits show that most QTL are population-specific. This population-specificity of QTL could be caused by different germplasms being used in the mapping populations, different planation conditions or different numbers of markers used for the studies. More work should be conducted on the other elite palm germplasms, to fully detect the loci for oil yield related traits for MAS in oil palm improvement.

### DNA markers associated with oil content and QTL combinations for improving oil content

Many studies have shown that marker-based strategies of QTL pyramiding are effective and practicable in breeding programs [37, 38]. Therefore, it is important to search for effective QTL associated with commercial traits. In our study, the genotypes with obvious negative and positive effects at four QTL associated with oil content traits were identified in an oil palm breeding population (Table 2 and Additional file 5: Table S5). Varying the combinations of genotypes of markers at these four QTL associated with O/B or O/DM showed that an increased number of QTL with positive effect, resulted in increased O/B (Fig. 5a), and a slight increase for the O/DM trait in the same breeding population (Fig. 5b). The obvious dosage effect showed that pyramiding these two O/B QTL and one or two of the O/DM QTL has great potential for oil palm breeding. Oil yield relative traits are complex and often controlled by large numbers of genetic loci with small additive effect [39], hence combining multiple QTL with beneficial genotypes in an individual can be an effective strategy of oil yield improvement in palm breeding.

Commercial seed production comes from crosses between phenotypically elite *Dura* and *Pisifera* trees, which produce higher yield *Tenera* trees for commercial plantation. MAS also can be used to identify the better *Dura* and *Tenera* individuals for the candidate of parents and tissue culturing, containing as many QTL as possible, in the early growth stage of the palms, instead of waiting till they are old enough for their yields to be measured. This reduces the generational interval and accelerates the genetic gain. Another way that MAS improves the efficiency of oil palm breeding is in the selection of *Dura* and parental *Pisifera* trees. The *Pisifera*, due to being female-sterile, and thus having no oil yield, were normally selected based on progeny tests, such as combining ability or heritability analysis by random choice, which need long, complicated selection cycles in palm breeding. MAS could be used to identify elite *Pisifera* trees carrying beneficial QTL, thus reducing the need for progeny tests. In our study, the beneficial genotypes of markers at QTL associated with oil content traits were identified in a breeding population. The latter was derived from a cross between an elite Deli *Dura* and an elite Ghana *Pisifera*, two parents which were used widely in the breeding program. Therefore, there is a great potential to pyramid these QTL in the breeding populations with similar genetic background as our mapping population. However, the effect of the QTL was based on statistical calculations in a particular breeding population, and the genetic background also has a great influence on the effect of QTL [40]. Hence, we need to verify the effectiveness of these QTL in other breeding populations with different genetic backgrounds from our population.

## Conclusions

We constructed a high-density linkage map with 1480 DNA markers. This map will provide a basis for fine mapping of QTL and improve the assembly of the genome of *Elaeis guineensis*. Four QTL for oil content traits and DNA markers associated with these traits were identified in a breeding population. The QTL combinations have the potential to accelerate the improvement of oil content traits in our breeding population by MAS. Further verification of the detected QTL for oil content traits in other genetic backgrounds and plantation conditions is required to examine whether the QTL detected in this study can be used in MAS in other palm populations.

## Methods

### Plant materials

All plant materials were derived from a cross between an elite heterozygous Deli *Dura* tree and a heterozygous Ghana *Pisifera* tree from the oil palm breeding program of Wilmar International Plantation. A total of 177 F_1_ *Tenera* progenies were planted at a plantation field at the same time in 2006 and managed under the same conditions in Indonesia. The field arrangement of the populations was carried out following the standard protocol of Wilmar Plantation in Indonesia. This cross was used to assess the oil yield traits.

### Phenotypic traits recoding

Oil to bunch content (O/B) was individually measured according to the standard protocol [1, 41] and recorded over three harvest periods (2011–2013). The oil to dry mesocarp content (O/DM) was measured according to the standard protocol [1, 41] and recorded for two harvest periods from six to seven years after planting (2012–2013). The oil content traits data from each year and arithmetic means for each individual were used for analysis of variance (ANOVA). The analysis of value distributions was performed with Excel 2013 (Microsoft).

### Genotyping microsatellites

A total of 177 oil palm progenies and two parental samples were available for genotyping analysis. Leaf samples (the leaf tips) were collected from individual palms and stored at −80 °C until DNA extraction. DNA was extracted and purified from leaf samples using the DNeasy Plant Mini kit (Qiagen, Germany). Genomic DNA quality was examined on 1.0% agarose gels, and quantification was conducted using Nanodrop 2000 (Nanodrop, DE, USA).

Microsatellite markers were developed using an enrichment method described in our previous paper [15, 42]. A total of 150 microsatellite markers (Additional file 4: Table S3) were selected based on a published linkage map [15], distributed in a wide coverage within and across all the 16 linkage groups. The forward primers for each microsatellite were labelled with either 6-FAM or HEX fluorescent dye. PCR reaction for each sample consisted of 10 ng of genomic DNA, 2.5 units of Taq-polymerase, 1× PCR buffer containing 1.5 mM MgCl_2_, 0.2 μM dNTPs, and 50 nM of each primer. PCR was performed on the thermal cycling machine (BioRad, CA, USA) under the following conditions: 3 min denaturation at 94 °C, 35 cycles of 30 s at 94 °C, 30 s at 52–58 °C and 45 s at 72 °C, and a final extension at 72 °C for 8 min. Then the PCR products were analysed on an ABI3730x1 DNA sequencer (Applied Biosystems, Foster City, USA). The allele sizes were analysed against the ROX-500 standard (Applied Biosystems, Foster City, USA) using GeneMapper 4.1 (Applied Biosystems, Foster City, USA). Then the data were used for genotype analysis for parental palms and their F_1_ palm progenies.

### RAD library preparation and sequencing

A total of 177 F_1_ palm progenies derived from a cross between *Dura* and *Pisifera*, and the two parent trees, were used for constructing RAD libraries. The genomic DNA concentration for the RAD libraries was measured by Infinite® M1000 PRO plate reader (Tecan, Männedorf, Switzerland) using Qubit® dsDNA HS Assay Kit (Life Technologies, USA). RAD libraries were constructed using the double-digest RADseq method with some modifications [9] as described in the previous study [30]. A total of 200 ng of genomic DNA from each sample was digested with 20 units of restriction enzymes *Pst*I-HF and *Msp*I (New England Biolabs, USA) at 37 °C for 3 h. Then, the DNA fragments were examined by electrophoresis on a 2% agarose gel before ligation with barcoded adaptors [9]. The ligation products were pooled, followed by the size selection of 300–500 bp using Pipin Prep (Sage Science, USA), and then a clean-up using QIAquick PCR Purification Kit (Qiagen, Germany). The selected fragments of libraries were PCR amplified using Phusion® High-Fidelity DNA Polymerase (New England Biolabs, USA), followed by the second library clean-up using QIAquick PCR Purification Kit (Qiagen, Germany). Quantification of libraries was performed using KAPA Library Quantification Kits (Kapa Biosystems, USA) by qPCR in the MyiQ Thermal Cycler (Bio-Rad Laboratories, Hercules, CA, USA). Finally, the libraries were sequenced on a NextSeq 500 platform (Illumina, San Diego, CA, USA) to generate paired-end raw reads of 2 × 150 bp in length.

### SNP identifying and genotyping

The raw reads were processed by the program *process_radtags* in Stacks package (version 1.21) [29] to remove low quality reads and any uncalled base, with a final step of de-multiplexing to assign clean reads to each sample. In order to reduce the sequencing errors at the end of each read, all the clean reads were trimmed to 125 bp. The cleaned reads were firstly aligned against the reference genome of oil palm [18] using the program BWA-MEM algorithm with default parameters. The alignment products of each individual were further used for stacks assembly and catalogue construction using program *pstacks* (−m, 3) and *cstacks* in Stacks package. The assembled stacks of each sample were matched to a catalogue constructed using parental samples to discover SNPs using *sstacks*. The program *genotypes* was used to call SNPs across progeny. For SNP calling, a minimum of 20 times coverage was applied in two parents, and a minimum of five times sequence coverage was applied for each sample. Any discovered SNP with more than 20% missing data in both genotypes and individuals were removed from further analysis, which finally left 153 progeny of high level data quality for further analysis.

### Genetic linkage map construction, unassembled scaffolds mapping and QTL analysis

The genotypic data of SSR and SNP markers for 153 F_1_ progeny palms derived from crossing *Dura* and *Pisifera* were used to construct genetic linkage maps with the software JoinMap 4.1 [43] and the genetic maps were drawn using the software MapChart V2.2 [44]. The markers with significant segregation distortion (*P* < 0.001) and markers having more than 10% missing data were excluded. Then the genotype data were grouped at LOD ≥ 4. Next, 18 nodes were selected to create groups for calculating the genetic linkage groups. The linkage groups were calculated and markers ordered based on the regression mapping algorithm. The Kosambi mapping function was used to estimate the map distance. The linkage groups for the mapping population were numbered in correspondence to the map published based on the anchor markers and genomic sequence [13, 18].

The genome sequence of oil palm *Elaeis guineensis* [18], was used as reference sequence for SNP genotyping in our study. It was mostly assigned into the 16 chromosomes of oil palm, but part of the genomic scaffolds is still unmapped on the chromosomes. Sequence mapping of the RAD loci from the high-throughput genetic map were assigned into 16 chromosomes. Using this genetic map information, the unassembled scaffolds were assigned into the 16 corresponding chromosomes.

The QTL detection was performed using the software MapQTL 6 [45]. The Interval mapping (IM) method was performed with the algorithm of regression, mapping step size 1.0 cM, and a maximum number of five neighbouring markers. To declare the presence of a significant or suggestive QTL, the threshold LOD values were estimated at the genome-wide (GW), and chromosome wide levels (5% of overall error level for both cases) by the permutation test (1000 permutations). The Kruskal-Wallis (K-W) test was performed to detect significant marker-trait associations at *P* < 0.05. The confidence interval of each QTL was determined by the LOD threshold value in a permutation test (*P* < 0.05). The proportion of phenotypic variance explained (PVE) by a single QTL was calculated by the square of the partial correlation coefficient (*R* ^*2*^) using the MapQTL software. The name for each QTL comprised of the trait, the linkage group, and a number to uniquely identify the QTL within the linkage group. For example, *Qoil_bunch_1.1* represents the first QTL for oil to bunch located on linkage group LG1 in oil palm.

## Additional files

Additional file 1: Table S1. Values of individual phenotypic traits, including means, ranges and coefficients of variation in the population of *Dura* × *Pisifera. (DOCX 13 kb)*

Additional file 2: Table S2. ANOVA for the phenotypic data of oil content traits in an oil palm breeding population of *Dura* × *Pisifera. (DOCX 19 kb)*

Additional file 3: Fig. S1. Distribution of phenotypic data (averaged over all periods) recorded in an oil palm breeding population used for QTL mapping. (JPEG 75 kb)

Additional file 4: Table S3. All the RAD markers and SSR markers with flanking sequences used in this study. **Table S4**. Mapping genome sequence of unknown chromosome in *Elaeis guineensis*, Jacq. (XLSX 182 kb)

Additional file 5: Table S5. Effects of marker genotypes at loci associated with oil content in a *Dura* × *Pisifera* breeding population. (DOCX 13 kb)

## Abbreviations

AFLP: Amplified fragment length polymorphisms
GBS: Genotyping by sequencing
GS: Genomic selection
GWAS: Genome-wide association study
LG: Linkage group
MAS: Marker-assisted selection
NGS: Next generation sequencing
O/B: Oil to bunch content (%)
O/DM: Oil to dry mesocarp content (%)
QTL: Quantitative trait loci
RAD-seq: Restriction-site-associated DNA sequencing
RAPD: Random amplified polymorphic DNA
SNP: Single nucleotide polymorphisms
SSR: Simple sequence repeats
