# Supplementary material for: Genome-wide identification of markers for selecting higher oil content in oil palm
Source: BMC Plant Biol. 2017 May 30;17:93. doi: 10.1186/s12870-017-1045-z (PMC5450198; doi:10.1186/s12870-017-1045-z)
Supplement: Supplementary file 1 — Values of individual phenotypic traits, including means, ranges and coefficients of variation in the population of Dura × Pisifera. (DOCX 13 kb) [file 12870_2017_1045_MOESM1_ESM.docx]

**Table S1 Values of individual phenotypic traits, including means, ranges and coefficients of variation in the population of *Dura* × *Pisifera***

| Trait | Harvest Period | Mean (n=153) | Range | Variance | S.E. Mean | Std Dev | CV (%) |
| --- | --- | --- | --- | --- | --- | --- | --- |
| O/B |  |  |  |  |  |  |  |
|  | 1^st^ period | 30.08 | 21.65-35.46 | 7.94 | 0.23 | 2.82 | 9.4 |
|  | 2^nd^ period | 29.88 | 21.07-37.35 | 11.19 | 0.29 | 3.34 | 11.2 |
|  | 3^rd^ period | 30.98 | 22.61-44.80 | 12.47 | 0.38 | 3.53 | 11.4 |
|  | average | 30.18 | 22.98-35.89 | 6.69 | 0.21 | 2.59 | 8.6 |
| O/DM |  |  |  |  |  |  |  |
|  | 1^st^ period | 79.45 | 68.75-87.38 | 10.1 | 0.26 | 3.18 | 4.0 |
|  | 2^nd^ period | 79.04 | 65.13-85.54 | 14.32 | 0.46 | 3.78 | 4.8 |
|  | average | 79.23 | 68.75-86.30 | 7.97 | 0.23 | 2.82 | 3.6 |

CV: coefficient of variation, Harvest periods: 1^st^ period in July, 2011, 2^nd^ period in July, 2012, 3^rd^ period in July, 2013
