# Supplementary material for: Genome-wide identification of markers for selecting higher oil content in oil palm
Source: BMC Plant Biol. 2017 May 30;17:93. doi: 10.1186/s12870-017-1045-z (PMC5450198; doi:10.1186/s12870-017-1045-z)
Supplement: Supplementary file 2 — ANOVA for the phenotypic data of oil content traits in an oil palm breeding population of Dura × Pisifera. (DOCX 19 kb) [file 12870_2017_1045_MOESM2_ESM.docx]

**Table S2 ANOVA for the phenotypic data of oil content traits in an oil palm breeding population of *Dura* × *Pisifera***

| Trait | Sources | df | SS | MS | F | *P value* |
| --- | --- | --- | --- | --- | --- | --- |
| O/B | Individuals | 152 | 3051.58 | 20.08 | 9.45 | <0.001 |
|  | Periods | 2 | 105.27 | 52.64 | 24.78 | <0.001 |
|  | Error | 304 | 645.85 | 2.12 |  |  |
|  | Total | 458 | 3802.71 |  |  |  |
| O/DM | Individuals | 152 | 2423.19 | 15.94 | 46.72 | <0.001 |
|  | Periods | 1 | 12.44 | 12.44 | 36.44 | <0.001 |
|  | Error | 152 | 51.87 | 0.34 |  |  |
|  | Total | 305 | 2487.5 |  |  |  |

O/B: oil to bunch (%), O/DM: oil to dry mesocarp (%)
