# Supplementary figures and images for: Genome-wide identification of markers for selecting higher oil content in oil palm
Source: BMC Plant Biol. 2017 May 30;17:93. doi: 10.1186/s12870-017-1045-z (PMC5450198; doi:10.1186/s12870-017-1045-z)

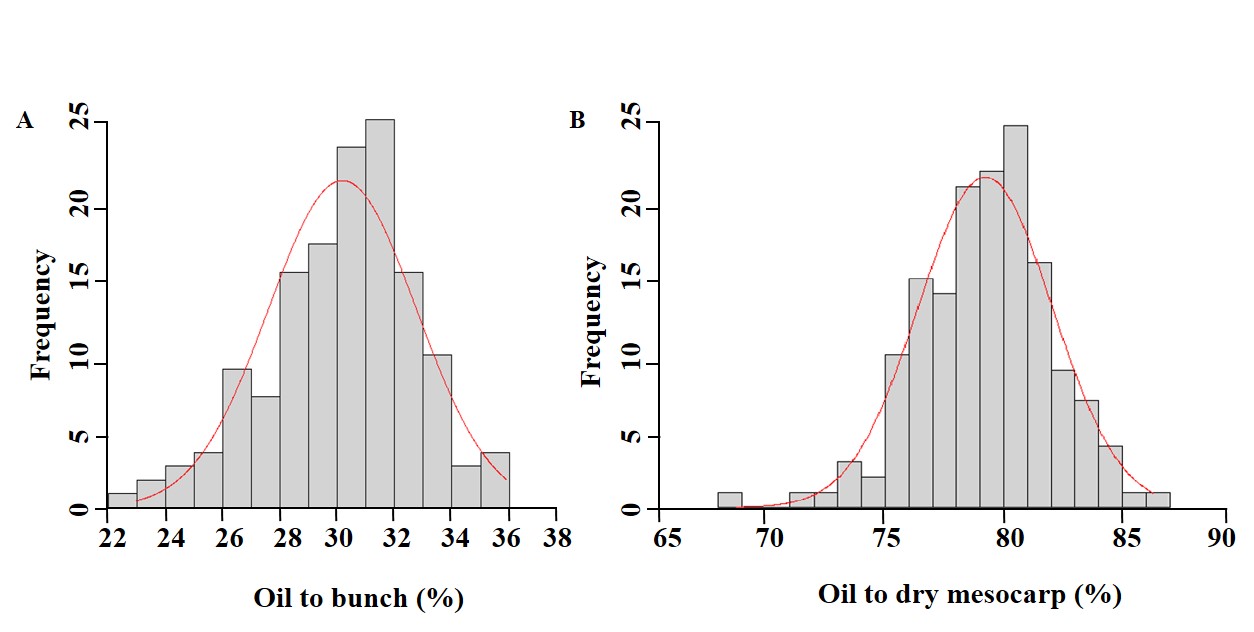

Supplement: Supplementary file 3 — Distribution of phenotypic data (averaged over all periods) recorded in an oil palm breeding population used for QTL mapping. (JPEG 75 kb) [file 12870_2017_1045_MOESM3_ESM.jpg]
