# Supplementary material for: Genome-wide identification of markers for selecting higher oil content in oil palm
Source: BMC Plant Biol. 2017 May 30;17:93. doi: 10.1186/s12870-017-1045-z (PMC5450198; doi:10.1186/s12870-017-1045-z)
Supplement: Supplementary file 5 — Effects of marker genotypes at loci associated with oil content in a Dura × Pisifera breeding population. (DOCX 13 kb) [file 12870_2017_1045_MOESM5_ESM.docx]

**Table S5 Effects of marker genotypes at loci associated with oil content in a *Dura* × *Pisifera* breeding population**

| Locus | Beneficial | | | |  | Non-beneficial | | |
| --- | --- | --- | --- | --- | --- | --- | --- | --- |
|  | Genotype | Frequency of genotype (%) | Average O/B (%) | Average O/DM (%) |  | Genotype | Average O/B (%) | Average O/DM (%) |
| *Qoil_bunch_1.1* |  |  |  |  |  |  |  |  |
| *EgSNP49153* | TT | 65.77 | 30.64 | - |  | AT | 29.47 | - |
| *EgSNP49181* | CC and C/T | 65.77 | 30.64 | - |  | TT | 29.56 | - |
|  |  |  |  |  |  |  |  |  |
| *Qoil_bunch_8.1* |  |  |  |  |  |  |  |  |
| *EgSNP5169* | GG | 50.32 | 30.8 | - |  | AG | 29.54 | - |
| *EgSNP61696* | GG | 51.7 | 30.9 | - |  | CG | 29.46 | - |
|  |  |  |  |  |  |  |  |  |
| *Qoil_mesocarp_8.1* |  |  |  |  |  |  |  |  |
| *Eg0129* | 203/196 bp | 56.21 | - | 79.26 |  | 203/203 bp |  | 78.91 |
| *Eg0985* | 227/221 bp | 56.2 | - | 79.57 |  | 227/227 bp |  | 78.79 |
|  |  |  |  |  |  |  |  |  |
| *Qoil_mesocarp_10.1* |  |  |  |  |  |  |  |  |
| *EgSNP40659* | C/T | 53.69 | - | 79.28 |  |  |  | 78.97 |
